# Autobiographical memory in contact tracing: evidence from the COVID-19 pandemic

**DOI:** 10.3389/fpsyg.2023.1244568

**Published:** 2023-11-24

**Authors:** Clelia Rossi-Arnaud, Serena Mastroberardino, Pietro Spataro, Alessandro Santirocchi, Federica Alessi, Aicia Naser, Maria Chiara Pesola, Vincenzo Cestari

**Affiliations:** ^1^Department of Psychology, Sapienza University of Rome, Rome, Italy; ^2^Department of Philosophy, Social Sciences and Education, University of Perugia, Perugia, Italy; ^3^Faculty of Society and Communication, University of the System of the Italian Chambers of Commerce, Rome, Italy

**Keywords:** contact tracing, autobiographical memory, COVID-19, recollection, lockdown

## Abstract

**Introduction:**

The recent COVID-19 pandemic has compelled various governments to trace all contacts of a confirmed case, as well as to identify the locations visited by infected individuals. This task, that requires the activation of our autobiographical memories, can make a difference in the spread of the contagion and was based primarily on telephone interviews with infected people. In this study, we examined whether participants were able to provide contact tracing information and whether their memories were influenced by salient events occurring during the initial phases of the pandemic.

**Methods:**

Participants were asked to fill in an online standardized form in which they recounted every day of the 2 weeks before, reporting as much information as possible. The time period selected included, among other things, the day on which the Italian government issued the decree initiating the COVID-19 lockdown. The task was completed twice, the first time relying solely on their memory, and the second time using external aids (diaries, mobile phones etc.). Reports were then coded using a scheme that segmented accounts into informational details, divided into two broad categories, internal and external.

**Results:**

Our findings showed that (i) the use of external aids was effective only when participants had to recall the day furthest away or if to-be-recalled events have low distinctiveness, and (ii) memories of internal details were recalled better than memories of external details. Participants were overall accurate and reported a large amount of information about people and places. However, because of the connection with key pandemic-related events, the effect was somewhat stronger on specific days (e.g., the day in which the lockdown was announced).

**Discussion:**

The results of this work could provide a useful tool for improving the design of contact tracing procedures in the event of an unwanted future public health crisis caused by a highly infectious agent.

## 1 Introduction

On 11 March 2020, Dr. Tedros Adhanom Ghebreyesus, WHO Director-General, declared at a press conference that the OMS “made the assessment that COVID-19 can be characterized as a pandemic.” Since then, attempts have been made in countries around the world to contain this virus, which is characterized by its very high contagiousness and late onset of symptoms, making it particularly insidious. The cost in terms of human lives has been very high and governments had to take emergency initiatives to stem the contagion by limiting contact between people. After an initial phase, in which lockdown appeared to be the only possible solution (although not applicable in the long term), other strategies were implemented. Of these, social distancing and contact tracing are undoubtedly the ones that have been most widespread in different countries around the world.

Contact tracing (CT) is a commonly used practice in virus spread control. Before the end of 2019, however, few people were familiar with this practice that implies remembering people and places visited during a specific time period. From this perspective, CT can thus be defined as a true memory task. Specifically, CT involves a particular type of memory, not just of past episodes, but more of an autobiographical nature.

Autobiographical memory refers to memory for one’s personal history (Robinson, 1976). Compared to episodic memory, that requires participants to recall what, when and where a specific event occurred, autobiographical memory is more related to autonoetic awareness (Tulving, 2002), but also to mental time travel and a sense of self (Conway and Williams, 2008). Autobiographical memory is also more influenced by social and cultural variables and appears to serve more social and self-defining functions. Fivush (2011) proposed that autobiographical memory can be distinguished from episodic memory since the former involves autonoetic consciousness, links past events into a personal history related to the past (Habermas and Bluck, 2000; McAdams, 2001) and guides past and future behavior (Bluck and Alea, 2002; Fivush et al., 2003). Autobiographical memory may be therefore considered as a biography of the self, a dynamic set of single or extended events, life periods, recurring experiences, which are organized in such a way as to create a coherent system of the self that gives us back the meaning of our existence (Conway et al., 2004; McAdams, 2008; Fivush, 2010). Thus, with respect to CT, it seems that when a person has to report on elements of a particular period of his or her recent life, we can assume that autobiographical memory is most involved in this task.

An interesting approach to study CT has been proposed by Garry et al. (2021) who view CT as a witness memory task, that is certainly related to autobiographical memory, but in a forensic context. When interviewing infected people about where they have been and who they met during a specific period of time, a number of challenges arise. We all have lives full of social encounters and places that we go to in our daily routines, and sometimes people tend to omit details about a specific event because one unconsciously prioritizes some information to the detriment of others (Mather and Sutherland, 2011), and therefore fails to recognize how relevant they may be for CT. Another issue is related to our ability to report specific details about an event. For example, one may report a visit to a shop and the interactions with other people without being able to recall how long it lasted (Grondin, 2010; Ravichandran-Schmidt and Hass, 2022) or the names of the people encountered (Cohen, 1990; Fawcett and Hulbert, 2020). In terms of CT this is a severe limitation as it makes it impossible to estimate exposure to infection and to trace potentially infected individuals. Our memory is also susceptible to distortions (see Schacter et al., 2011). We tend to be confused about the source of our memory (Johnson et al., 1993) and we rely on past experience and schematic knowledge (Graesser and Nakamura, 1982; Barry and Love, 2023). However, there are also other factors to consider. For example, many COVID-19 patients are affected by concurrent cognitive deficits (Mosser and Evans, 2019; Hosp et al., 2021), that make it more difficult for them to perform a CT task.

To overcome human limitations on CT, the available technology may provide a useful help. In a recent review on automated and partly automated CT, Braithwaite et al. (2020) analyzed studies on the effectiveness of tools such as contact tracing apps versus partly automated systems and found that the former was less effective in predicting contact identification and, consequently, in reducing viral transmission. According to the authors, this may be because, to be effective, such automated contact tracing applications need to be used by a high proportion of the population (56–90%; see Xia and Lee, 2020; Kim and Paul, 2021). In Italy, however, the Immuni app, launched by the Ministry of Health, had been downloaded by 21,882,502 people by 31 December 2022, far less than half of Italy’s population, and only 92,073 people had registered their positivity on the app, allowing them to send a message to 196,114 contacts. For the same period of time, data collected by the Johns Hopkins University Center for Systems Science and Engineering (JHU CSSE) from various sources (WHO; ECDC; US CDC and others) reported a number of infections of 25.143.705 for the Italian population. It is interesting to note that, according to Braithwaite et al. (2020), partly automated contact tracing (i.e., involving some level of automation in the process but not in the use of devices that gathers contact data) was instead reported to be more effective as compared to manual contact tracing. This may be due to the fact that partly automated contact tracing implies an active participation by the infected person combined with a less invasive use of technology, which makes it more acceptable to those who have to give up their privacy in order to trace possible infected persons.

A recent study by Evans et al. (2022) tried to compare the efficacy of a psychologically based contact tracing interview protocol to a control protocol that emulated current practices under both interviewer-led and self-led modalities. Authors compared participants’ performance on CT using a computerized version of the Enhanced Cognitive Interview, a well-established protocol that has been proved to enhance recall and accuracy (Fisher and Geiselman, 1992), versus a control protocol where participants received instructions to report their contacts and places visited during the last 6 days. Information was gathered either with an interviewer-led call or with a self-led online survey. In the latter part of the interview participants were encouraged to use their phone or calendar to prompt further recall and provide additional details about the reported contacts and locations. The Enhanced Cognitive protocol took significantly longer to complete than the control protocol but participants in this condition provided more information, as predicted by the literature (see Memon et al., 2010 for a meta-analysis). Moreover, there was no difference between interview modalities, showing that self-led online survey interviews are equally effective as interviewer-led ones.

Starting from this background, the aim of the present study was to investigate whether participants were able to provide contact tracing information when they had to fill in a Google form to indicate the activities they had undertaken, the places they had visited and the encounters they had made in the 2 weeks prior to the administration (from the 12th of March to the 27th of February 2020). To further explore potential advantages associated with the use of technological support, participants were asked to recall the events with or without external aids (diaries, mobile phones). The selected period included, among other things, the day on which the Italian government issued the decree initiating the COVID-19 lockdown and the day in which university classes were suspended. Each report was then coded using a scheme derived by Levine et al. (2002), that segmented accounts into informational bits or details, divided into two broad categories, internal and external. Internal details (which included the sub-categories event, place, time, perceptual, emotion/though) can be defined as those that were directly referred to the main event described by the participant, were specific in terms of time and place, and were considered to reflect episodic recollection. External details (which included the subcategories external events, semantic, repetition and other), in contrast, can be defined as those that pertained to specific autobiographical events other than the main defined internal event (i.e., factual information or extended events that did not require the recollection of a specific time and place: Levine et al., 2002). A secondary aim was to investigate whether individual differences in anxiety influenced the number of events recalled in the autobiographical forms. To this purpose, we also asked participants to fill in the State–Trait Anxiety Inventory (STAI-Y: Spielberger, 1970; Spielberger et al., 1983), which measures both state and trait anxiety.

Our detailed predictions were as follows. First, we expected to find a recency effect, such that participants should recall more events from the couple of days immediately preceding the beginning of the administration than for other days. Standard recency and primacy effects have been indeed reported in recent studies investigating autobiographical memory for 2020 and its relation to COVID-19 events (Rouhani et al., 2023). Related to this, we also expected that the recall performance should be higher for days characterized by salient events, such as the issue of the decree that suspended all classes. This expectation is supported by a large body of research indicating that flashbulb memories are retained and remembered particularly well, due to their association with public, emotionally charged events (i.e., Brown and Kulik, 1977, but see Neisser and Harsch, 1992; Hirst and Phelps, 2016). Applying this knowledge to the COVID-19 context, Öner et al. (2023) found that national and global events occurring during the onset and course of the pandemic were shared across many counties, laying the foundations of emergent collective memories. Similarly, Rouhani et al. (2023) showed that there was a pronounced peak of autobiographical memory for March 2020, aligning with the outburst of COVID-19 and the announcement of lockdowns, and concluded that unexpected, high-impact transitions may influence the storage of autobiographical memories (see Brown, 2021, for a theoretical model). Conversely, we expected that recall should be lower for days following the beginning of the lockdown, because of the strong reduction in people’s mobility. Second, in line with previous studies, we predicted that participants should recall more details from internal than external categories (Levine et al., 2002). When comparing different conditions, we predicted that the use of support tools should increase the number of events recalled, especially on the most distant days. Lastly, regarding the impact of individual differences in state and trait anxiety on memory performance, different expectations could be put forward. On the one hand, there is evidence indicating that high levels of anxiety and chronic stress may hamper the recall of autobiographical memories (Hallford et al., 2018, 2019). On the other hand, the phenomenon known as ‘emotionally enhanced memory’ suggest that positive and negative events tend to be remembered better than neutral events (Williams et al., 2022). In line with this expectation, Rouhani et al. (2023) found that stronger negative affect predicted enhanced autobiographical memory.

## 2 Method

### 2.1 Participants

A total of 44 students from the Department of Psychology of the University Sapienza of Rome were recruited in the presented study and participated voluntarily, of whom 38 completed the procedure and 6 dropped out (age range: 21–34 years, *M* = 23.34, *SD* = 2.43; 6 males). They were students enrolled in a Psychology course held by the project leader (Prof. Rossi-Arnaud), who was taking online classes at the time of testing. Twenty-three of them were off campus students and 15 of them went back to their hometown after the lockdown.

### 2.2 Materials

Three different questionnaires (Google forms) were used. The first one was a *personal data questionnaire* where participants had to report the following information: age, gender, if they were off-campus students, if they went back home before lockdown and if they lived with their family, in a shared accommodation or alone. The second questionnaire was an *autobiographical memory form* in which they had to indicate backwards (starting from the day before that of completion up to 14 days before), for each day, what they remembered about the places they frequented and the movements they made (alone or in company), with an indication of the approximate time and the people present. For every day participants were asked to indicate a maximum of 10 events. In order to respect the anonymity of other persons involved in the event, these were indicated only by the initials of the name (e.g., G.); furthermore, for places frequented, in order to respect privacy, only the type of place (e.g., cinema) was to be indicated, but not the name; in the case of a bus journey, the bus number was not to be indicated (see examples below).

*Cinema from 6 to 8 p.m.: with G.B. and A.F.*

*Bus No XXX taken at XXX with a journey of about 20 min from the starting.*

*Lesson from 9 to 11 a.m. in Lecture Hall XX with K., L., P. and G.*

This form had to be filled in twice: the first completion was based on autobiographical memories alone (AM1), whereas for the second completion participants were asked to use external aids, such as diaries, mobile phones, calendars or the help of other people, and had to indicate the type of aid employed (AM2). All participants reported using their mobile phones as a source of information; only 14 participants reported using also diaries and social networks.

Finally, participants were asked to fill in the State–Trait Anxiety Inventory (STAI-Y: Spielberger, 1970; Spielberger et al., 1983), were debriefed and thanked for their participation. The entire procedure lasted around 60 min.

### 2.3 Procedure

Each student received an email with a description of the study and a link to the online informed consent form. If willing to participate, he/she had to provide a univocal code before entering the consent form. Once the signed consent was received from the participant, the links to the three different questionnaires (Google forms) were sent out. Participants used their code to fill in the questionnaires, to ensure anonymity. The study was approved by the Institutional Review Board (ethics committee) of the Department of Psychology (School of Medicine & Psychology) of Sapienza University (Rome, Italy) and all the procedures adhered to the principles of the Declaration of Helsinki.

### 2.4 Scoring of the autobiographical memory questionnaire and data analysis

Data were coded according to Levine et al. (2002) coding scheme. Specifically, each event was segmented into informational bits or details, defined as a unique occurrence or thought expressed as a grammatical section with a subject and predicate (e.g., “*I woke up at 8 a.m.*”). Events were divided into two broad categories, internal and external. The former included those related to the main event described by the participant, with a specific time and place, and were considered to reflect episodic reexperiencing. The latter were specific autobiographical events other than the internal ones. Internal details were further separated into five mutually exclusive categories: (a) event (i.e., happenings, individuals present, weather conditions, physical/emotional actions, or reactions in others); (b) place (localization of an event including the city, street, building, room, part of room); (c) time (year, season, month, day of the week, time of day); (d) perceptual (auditory, olfactory, tactile, taste, visual details, body position and duration) and (e) emotion/thought (emotional state, thoughts, implications). External details were divided into four mutually exclusive categories: (a) event (i.e., details from other experiences and incidents, external to the main event recalled); semantic (i.e., general knowledge or facts, ongoing events, extended states of being); repetition (i.e., repetition of already mentioned details) and other (i.e., metacognitive statements, editorializing).

**Figure 1:**
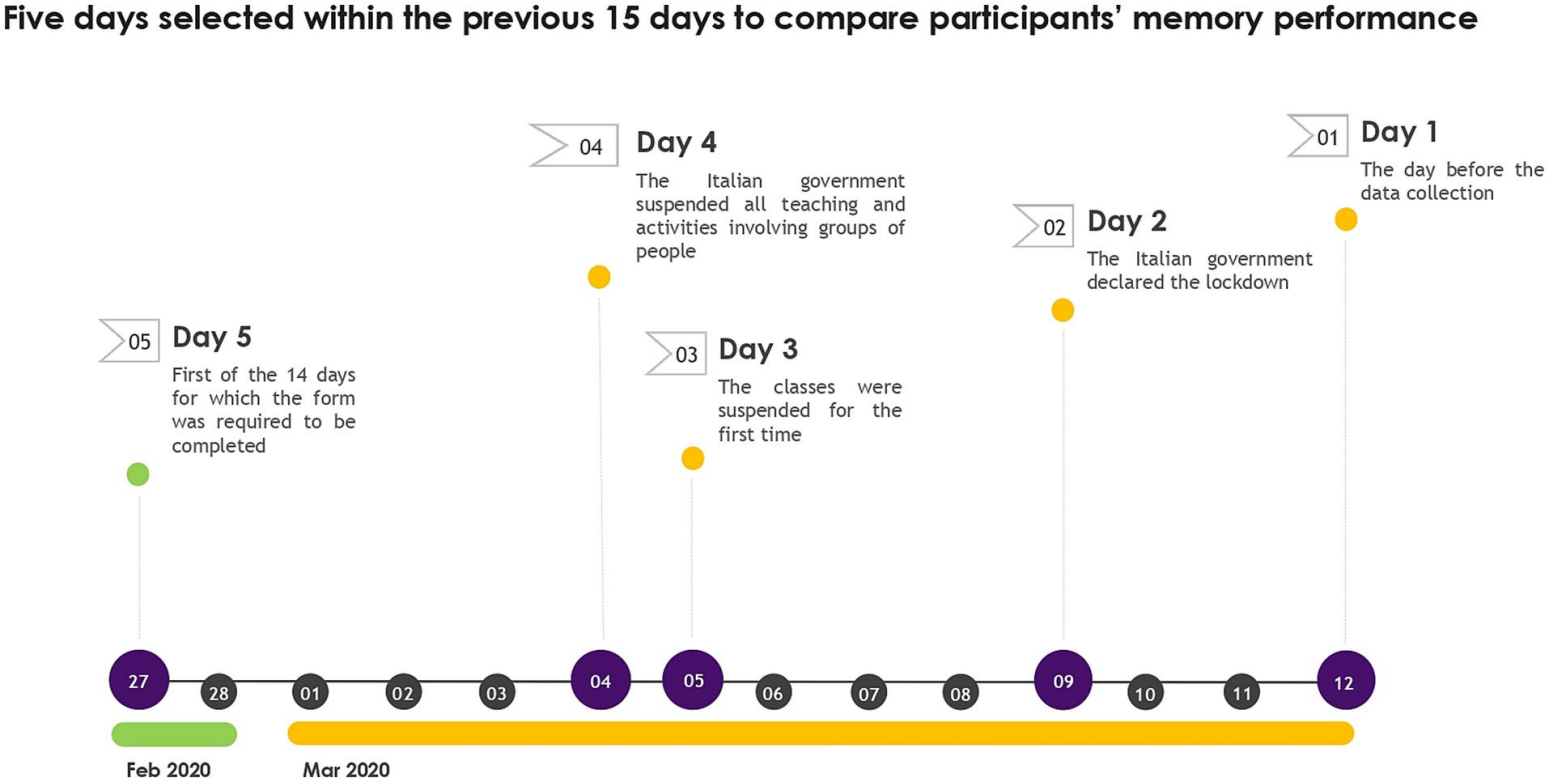
Timeline showing the five selected days, in the 15-day period prior to the collection date, on which participants’ memory performance was analyzed.

As our participants were instructed to report a maximum of 10 events per day and recalled very few details, we could not code for episodic richness; we instead opted for counting the raw number of details reported in the memory form. Although participants reported a maximum of 10 events per day for 14 days (from March, 12 to February, 27), five relevant days were selected to compare participants’ memory performance over time, namely: (a) March,12, 2020, the day before the data collection (henceforth “Day 1″); (b) March, 9, 2020, when the Italian government declared the lockdown (henceforth “Day 2″); (c) March, 5, 2020, when the classes were suppressed for the first time (henceforth “Day 3″); (d) March, 4, 2020, when the Italian government suspended all teaching and activities involving groups of people (henceforth “Day 4″); (e) February, 27, 2020, the first of the 14 days for which the form was required to be completed (henceforth “Day 5″). These 5 days were chosen because days 1 and 5 were the most recent and farthest away, while days 2, 3 and 4 represented important turning points in the lives of our participants (see Figure 1 for a timeline showing the days selected for analyses). Two trained raters were asked to code 20% of the memory forms. Interrater reliability was assessed with intraclass correlation (one-way random effects model; McGraw and Wong, 1996). Agreement was high for both internal details (0.96) and external details (0.89) and ranged between 0.87 and 0.95 for specific probes.

## 3 Results

### 3.1 Number of reported events

**Figure 2:**
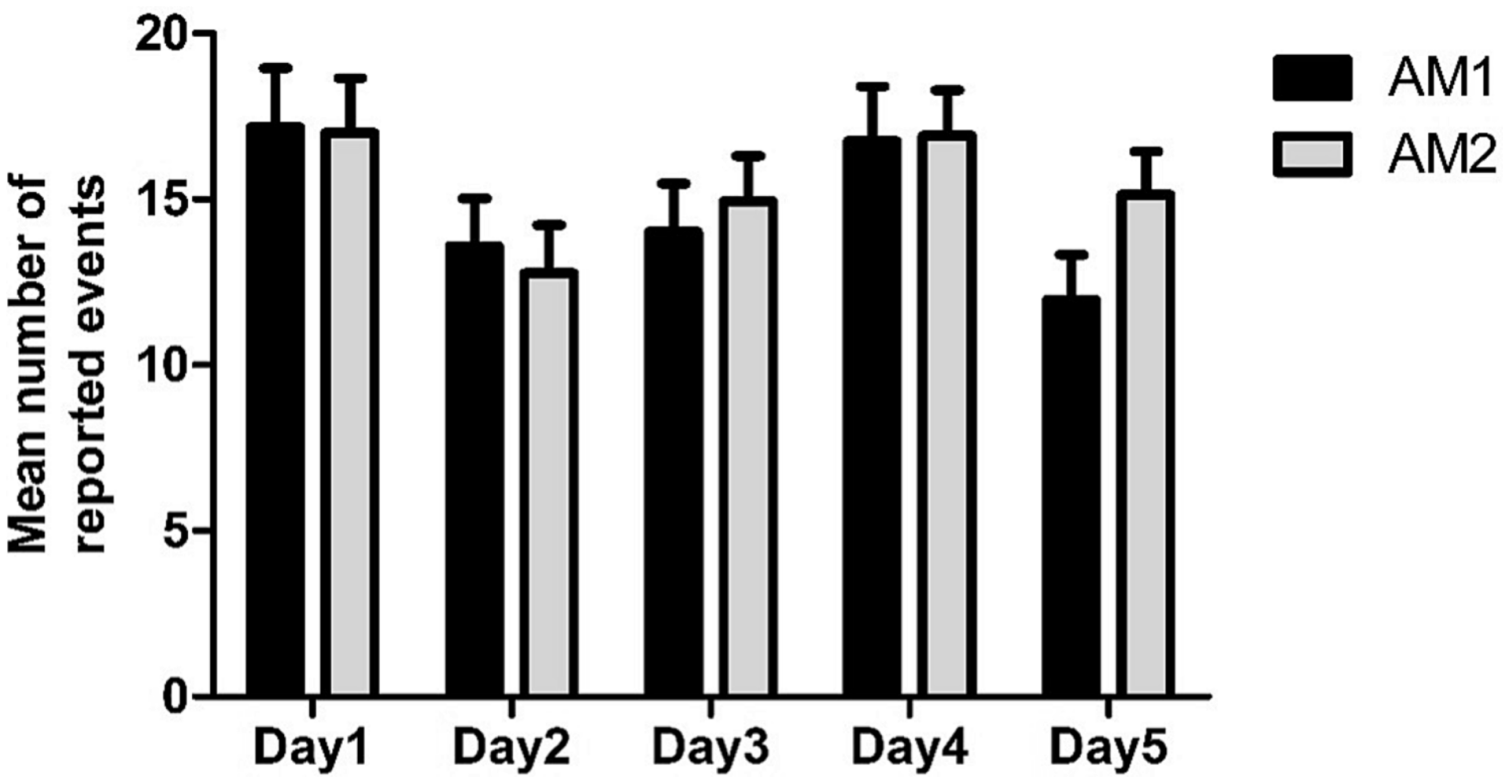
Means and SD for the total number of reported events, as a function of Condition and Time.

A 2 (Condition: AM1 vs. AM2) × 5 (Time: Day 1, Day 2, Day 3, Day 4, Day 5) repeated measure ANOVA was conducted on number of reported events (see Figure 2). A significant main effect was found for Time [*F*(4, 148) = 6.33, *p* < 0.001, η^2^_p_ = 0.15], together with a significant interaction between Condition and Time [*F*(4, 148) = 2.89, *p* = 0.024, η^2^_p_ = 0.07]. A follow-up analysis of simple effects revealed that the use of external aids improved memory only on Day 5 [*F*(1, 37) = 5.37, *p* = 0.026, η^2^_p_ = 0.13], but not on other days [all *F*(1, 37) < 2.51, *p* > 0.012]; moreover, the main effect of Time was significant in both the AM1 and AM2 conditions [*F*(4, 34) = 6.50, *p* = 0.001, η^2^_p_ = 0.43 and *F*(4, 34) = 4.75, *p* = 0.004, η^2^_p_ = 0.36, respectively]. In the AM1 condition, the post-hoc comparisons (with the Bonferroni correction) showed that participants reported significantly more events for the 12th of March (Day 1) as compared to Days 2, 3, and 5 (all *p*s < 0.007), which is conceivable since the former is the nearest day to be remembered (see Table 1 for means and SD). In addition, participants reported marginally more information for Day 4 (9th of March) as compared to Day 5 (*p* = 0.065). As for the AM2 condition a similar pattern of results was present, with participants reporting more events for Day 1 as compared to Days 2 and 5 (*p* = 0.001 and *p* = 0.053, respectively).

**Table 1:** Means and SD for number of reported details for each internal category.

Internal category	Time	AM1		AM2	
		Mean	SD	Mean	SD
Event					
	Day 1	9.03	5.86	9.87	5.7
	Day 2	7.26	4.89	6.76	4.92
	Day 3	7.31	5.05	7.36	4.71
	Day 4	7.71	5.03	7.66	4.5
	Day 5	5.45	3.96	6.84	3.69
Place					
	Day 1	1.47	1.81	1.31	1.27
	Day 2	1.63	1.44	1.53	1.3
	Day 3	2.26	1.92	2.63	1.67
	Day 4	3.36	2.74	3.28	2.24
	Day 5	2.63	2.36	3.21	2.66
Time					
	Day 1	5.15	3.84	5.31	3.44
	Day 2	3.79	2.67	3.63	2.68
	Day 3	3.47	2.72	3.92	2.63
	Day 4	4.02	2.65	4.52	2.41
	Day 5	2.87	2.47	4.02	2.56
Perceptual					
	Day 1	0.42	1.03	0.5	0.92
	Day 2	0.34	0.9	0.18	0.6
	Day 3	0.02	0.16	0.16	0.44
	Day 4	0.10	0.5	0.16	0.44
	Day 5	0.29	0.87	0.34	0.97
Emotion/Thoughts					
	Day 1	0.68	1.18	0.55	0.95
	Day 2	0.39	0.75	0.31	0.52
	Day 3	0.60	1.09	0.63	1.02
	Day 4	0.81	1.24	0.71	1.01
	Day 5	0.47	0.80	0.47	0.72

### 3.2 Internal versus external details

**Figure 3:**
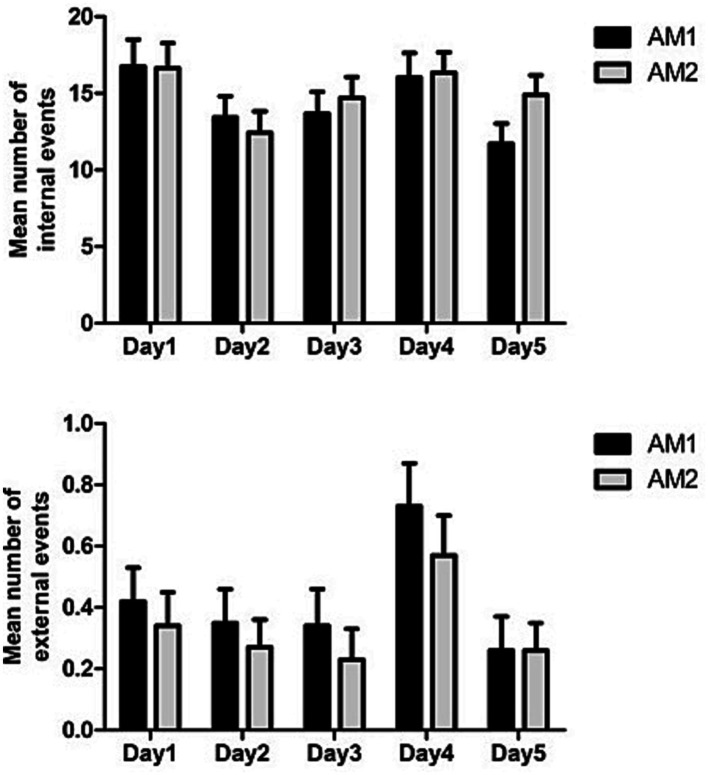
Means and SD for the total number of internal and external events, as a function of Condition and Time.

A second analysis was conducted on number of internal vs. external details provided for each day (see Figure 3). Specifically, a 2 (Condition: AM 1 vs. AM2) × 2 (Type of details: internal vs. external) × 5 (Time: Day 1; Day 2; Day 3; Day 4; Day 5) repeated measure ANOVA was conducted on number of details provided. The results showed significant main effects of Time [*F*(4, 148) = 14.00, *p* < 0.001, η^2^_p_ = 0.28] and Type of details [*F*(1, 37) = 216.00, *p* < 0.001, η^2^_p_ = 0.85], which were qualified by significant two-way interactions between Condition and Time [*F*(4, 148) = 3.43 *p* = 0.010, η^2^_p_ = 0.09] and between Type of details and Time [*F*(4, 148) = 14.34, *p* < 0.001, η^2^_p_ = 0.28], as well as by a significant three-way interaction between Condition, Type of details and Time [*F*(4, 148) = 3.45, *p* = 0.010, η^2^_p_ = 0.09]. A follow-up analysis of simple effects on the latter interaction revealed that: (a) the use of external aids improved only the recall of Internal details on Day 5 [*F*(1, 37) = 6.90, *p* = 0.012, η^2^_p_ = 0.16]; (b) more internal than external details were reported in all conditions [all *F*(1, 37)s > 78.96, *p* < 0.001]; and (c) the main effect of Time was significant for internal details in both the AM1 and AM2 conditions [*F*(4, 34) = 20.06, *p* < 0.001, η^2^_p_ = 0.70 and *F*(4, 34) = 22.70, *p* < 0.001, η^2^_p_ = 0.72, respectively], whereas it was marginal or non-significant for external details [*F*(4, 34) = 2.27, *p* = 0.082, η^2^_p_ = 0.21 in the AM1 condition and *F*(4, 34) = 1.46, *p* = 0.23, η^2^_p_ = 0.14 in the AM2 condition]. For internal details in the AM1 condition, the post-hoc pairwise comparisons (with Bonferroni correction) showed that participants recalled less details on Day 3 than on all other days (all *p*s < 0.008). Similarly, for internal details in the AM2 condition, participants recalled less details on Day 3 than on all other days (all *p*s < 0.005); in addition, in this condition, participants recalled more details on Day 1 than on Day 2 (*p* = 0.016).

### 3.3 Analysis of internal categories

A series of 4 (Time: Day 1; Day 2; Day 3; Day 4; Day 5) × 2 (Condition: AM 1 vs. AM2) repeated measure ANOVAs were performed on number of details belonging to each internal category (event, place, time, perceptual, emotion/thought). Table 1 reports means and standard deviations for all Internal categories.

Results showed a significant main effect of Time on number of recalled *events* [*F*(4, 148) = 4.86 *p* = 0.001, η^2^_p_ = 0.12], together with a significant interaction between Condition and Time [*F*(4, 148) = 2.70 *p* = 0.033, η^2^_p_ = 0.07]. A follow-up analysis of simple effects showed that the use of supportive instruments improved the recall of events on Day 5 [*F*(1, 37) = 5.01, *p* = 0.003, η^2^_p_ = 0.11], but not on Days 1–4 [all *F*(1, 37)s < 2.09, *p* > 0.15]. The same analysis indicated that the main effect of Time was significant in both the AM1 and AM2 conditions [*F*(4, 34) = 3.46, *p* = 0.018, η^2^_p_ = 0.29 and *F*(4, 34) = 3.30, *p* = 0.022, η^2^_p_ = 0.28, respectively]. The post-hoc comparisons (with Bonferroni correction) revealed that, in the AM1 condition, participants recalled less events on Day 5 than on Days 1 and 4 (*p* = 0.013 and *p* = 0.043, respectively). In the AM2 condition, participants recalled more events on Day 1 than on Day 2 (*p* = 0.007).

As for remembered *place* details, only a significant effect of Time was found [*F*(4, 148) = 8.52, *p* < 0.001, η^2^_p_ = 0.19]. The post-hoc comparisons (Fisher’s Least Significance Difference) indicated that participants recalled less place details on Day 1 than on Days 3, 4 and 5 (all *p*s < 0.047), and on Day 2 than on Days 4 and 5 (*p* = 0.004 and *p* = 0.036, respectively). These differences may be attributed to the fact that the restrictions imposed by the government affected participants’ mobility, forcing them to spend most of their time at home.

For remembered *time* details, significant main effects of Condition [*F*(1, 37) = 4.97, *p* = 0.032, η^2^_p_ = 0.11] and Time [*F*(4, 148) = 5.06, *p* = 0.001, η^2^_p_ = 0.12] were found, together with a significant two-way interaction [*F* (4, 148) = 3.20, *p* = 0.015, η^2^_p_ = 0.08]. A follow-up analysis of simple effects showed that the use of external aids improved the recall of time details on Day 5 [*F*(1, 37) = 11.02, *p* = 0.002, η^2^_p_ = 0.23], but not on Days 1–4 [all *F*(1, 37)s < 2.59, *p* > 0.11]. The same analysis indicated that the main effect of Time was significant in both the AM1 and AM2 conditions [*F*(4, 34) = 3.50, *p* = 0.017, η^2^_p_ = 0.29 and *F*(4, 34) = 3.56, *p* = 0.016, η^2^_p_ = 0.30, respectively]. The following post-hoc comparisons (with Bonferroni correction) highlighted that, in the AM1 condition, participants recalled more time details on Day 1 than on Days 3 and 5 (*p* = 0.030 and *p* = 0.014, respectively). In the AM2 condition, participants recalled more time details on Day 1 than on Day 2 (*p* = 0.008).

No other significant effects were found for the residual two categories (perceptual and emotion/thought).

### 3.4 Analyses of external categories

A series of 4 (Time: Day 1; Day 2; Day 3; Day 4; Day 5) × 2 (Condition: AM 1 vs. AM2) repeated measure ANOVAs were likewise performed on the number of details belonging to each external category (external event, semantic, repetition, other; see Table 2).

**Table 2:** Mean and SD for number of external details reported.

External category	Time	AM1		AM2	
		Mean	SD	Mean	SD
Event					
	Day 1	0.21	0.47	0.18	0.51
	Day 2	0.29	0.69	0.15	0.43
	Day 3	0.21	0.62	0.15	0.43
	Day 4	0.44	0.68	0.36	0.63
	Day 5	0.00	0.00	0.07	0.27
Semantic					
	Day 1	0.10	0.38	0.05	0.22
	Day 2	0.02	0.16	0.05	0.22
	Day 3	0.05	0.22	0.05	0.22
	Day 4	0.10	0.31	0.07	0.27
	Day 5	0.00	0.00	0.02	0.16
Repetition					
	Day 1	0.05	0.22	0.10	0.50
	Day 2	0.02	0.16	0.05	0.22
	Day 3	0.05	0.22	0.02	0.16
	Day 4	0.07	0.27	0.13	0.41
	Day 5	0.02	0.16	0.10	0.45
Other					
	Day 1	0.00	0.00	0.00	0.00
	Day 2	0.00	0.00	0.00	0.00
	Day 3	0.00	0.00	0.00	0.00
	Day 4	0.00	0.00	0.00	0.00
	Day 5	0.00	0.00	0.05	0.32

A significant main effect of Time was found only for the *event* category [*F*(4, 148) = 4.13, *p* = 0.003]. Specifically, participants reported less event details on Day 5 as compared to all other days (see Appendix Table A1 for means and SD). As for other External categories (repetition, semantic) no effects were found. The *Other* category was mentioned only by one participant twice and was therefore excluded from statistical analyses.

### 3.5 Anxiety as measured by the StaiY1 and Y2

Participants’ state and trait anxiety was measured using the State–Trait Anxiety Inventory (STAI-Y, Spielberger, 1970). Scoring of the scales highlighted that participants reported mild state (*M* = 44.46, *SD* = 9.38) and trait anxiety (*M* = 44.20, *SD* = 7.92) levels. To further investigate the impact of state and trait anxiety on participants’ performance we correlated the STAI-Y1 and STAI-Y2 scores with memory measures. No significant correlations were found in relation to memory measures.

## 4 Discussion

The aim of this study was to investigate whether participants were able to provide CT information by completing a standardized online form in which they were asked to recount each day of the 2 weeks prior to the study (from 12th March to 27th February 2020). Participants were instructed to report as much information as possible and to complete the form twice, the first time relying on recollection alone and the second time also using external aids (diaries, mobile phones etc.), to compare potential differences. The 2 weeks’ period included, among the other things, the day on which the Italian government issued the decree initiating the COVID-19 lockdown and the day in which classes were suspended. Each report was coded using a scheme derived by Levine et al. (2002), that segmented accounts into informational bits or details, divided into two broad categories, internal (which included the sub-categories event, place, time, perceptual, emotion/though) and external (which included the subcategories external events, semantic, repetition and other).

Our first expectation was that participants would recall more events from recent days compared to those further back. In agreement, the analysis of the overall number of details reported showed a significant recency effect, with participants recalling more events from Day 1 (the most recent day reported) than from other days. A previous study investigating autobiographical memory during the COVID-19 period found evidence of both primacy and recency effects (Rouhani et al., 2023). Specifically, when asked to report memories for the 2020 year on December of that year, participants recalled more events from the first month of the year (January, compared to February) and from the last month (November, compared to October). In this respect, our results replicated the recency effect using a much shorter period of time.

Participants in the AM1 condition reported more information on Day 4 than on Day 5 (the furthest day to be remembered). The difference was significant when analyzing the internal event category and marginally significant when analyzing the total number of details reported. These findings are consistent with our expectation that the recall performance should be higher for days characterized by events that were salient to participants. Specifically, the distinctiveness principle assumes that events will be well remembered to the extent that they are more distinct than competing events at the time of retrieval (Surprenant and Neath, 2009; Neath and Saint-Aubin, 2011). In our case, the events occurring on Day 4 were likely more distinctive than those occurring on Day 5, because the former was the day in which the Italian government issued the decree of suspension of all classes - an event which had important consequences on the lives of our participants. Convergent evidence comes from the afore-mentioned study by Rouhani et al. (2023), in which there was a strong bump in autobiographical memory for March 2020, which was characterized by the onset of COVID-19 emergency and lockdown announcements. Taken together, these results suggest that unprecedented collective events and transitions may shift personal narratives by modulating the distribution of autobiographical memories (Brown, 2021; Öner et al., 2023; Rouhani et al., 2023).

The distinctiveness principle may also be helpful for explaining the finding that the use of external aids, such as diaries, mobile phones, or calendars, was effective in enhancing memory for Day 5, but not for other days. The first 4 days of data analysis were notable for the significance of the events that defined them (Days 2, 3, and 4) or for being the most recent events experienced (Day 1); in contrast, recall of Day 5 events suffered because they were both the most distant in time and bore no particular relevance to participants’ lives. Thus, our data indicate that the use of external instruments may help CT especially when the to-be-recalled events have low distinctiveness. On the other hand, external aids did not increase the recall of internal and external information for Days 2, 3 and 4, likely because the association with early-phase pandemic-related events (which are well-retained, due to their high emotional salience, the wide media coverage and the impact of personal concerns about COVID-19: Cole et al., 2022; Rouhani et al., 2023) made these episodic memories particularly accessible and protected from decay.

When we analyzed more specifically the number of internal and external details provided by participants, it turned out that, in line with our expectations, internal details were recalled more often than external details. This is not a surprising result, since external categories tend to be poorly recalled in autobiographical accounts (see Levine et al., 2002). We also found that participants reported significantly less internal events on Day 3 than on all other days. This output is consistent with our predictions because Day 3 was the first day in which classes were suspended. It seems likely that most of the daily activities of our participants were consequently blocked, resulting in a paucity of events and encounters. Such an explanation is further bolstered by the finding that the recall of places was particularly poor on Days 1 and 2. These were indeed the days in which the lockdown decree came into force and participants were therefore confined to their homes. At the same time, the poor recall of internal events on Day 3 fits well with expectations since Day 3 occupies an intermediate position in the mental timeline tested in the present study. The recall of middle-list items is typically worse than the recall of items presented in the first positions (Tulving, 1972, 2002), and autobiographical memory appears to follow a similar principle - i.e., the recall of oldest events is worse than the recall of more recent events (Wagenaar, 1986).

Regarding the relation of autobiographical memory with anxiety, our results indicate that, although participants experienced mild levels of state and trait anxiety, the intensity of these feelings was unrelated to the number of internal and external events reported. As illustrated in the Introduction, previous evidence about this issue reached mixed conclusions. Some findings suggest that high levels of anxiety, being linked to chronic stress, should impair the recall of specific autobiographical details (Hallford et al., 2018, 2019). Autobiographical memory is thought to rely on generative search of self-related memories that are stored in a hierarchical structure (from abstract information on the self to specific events; Conway and Pleydell-Pearce, 2000). Anxiety may disrupt the efficacy of these generative processes in several ways – by reducing attention control, by focusing participants’ attention on threatening thoughts, by reducing their ability to filter out irrelevant stimuli and/or by reducing working memory capacity (Hallford et al., 2018). Other evidence, in contrast, suggest that, when it comes to deliberate recall, negatively-charged memories are retained and remembered better than neutral memories (LaBar and Cabeza, 2006; Rossi-Arnaud et al., 2018). Since direct retrieval contributes to autobiographical memory (Uzer et al., 2012; Markostamou et al., 2023), it can be inferred that events associated with high levels of anxiety and negative affect should be recalled better than events associated with low levels of anxiety. In this respect, our results suggest that high state and trait anxiety did not affect the number of autobiographical events reported. Several caveats should be noted, however. First, as mentioned above, we were unable to code data for episodic richness; thus, we could not exclude the possibility that anxiety may have significant effects on this variable. Second, most of the events that participants were asked to recall were highly salient and unusual, since they had never experienced a pandemic before. Flashbulb memories (defined as the recollection of particularly salient, surprising or consequential events) are known to engender more anxiety than common autobiographical memories (Conway et al., 2009) and may be therefore less sensitive to non-clinical variations of state or trait anxiety. Finally, we measured anxiety at the time of testing, but did not assess self-reported affect during the tested periods. This is relevant because Rouhani et al. (2023) found that the likelihood of retrieving a month was positively related to the degree of negative affect experienced during that month.

The present study has several limitations that must be acknowledged. First, our sample size was relatively small. Using G*Power3 (Faul et al., 2007), we ran a sensitivity analysis and estimated that, with a power of 0.80, our sample was sufficient to detect an effect size of *d* = 0.18 and larger in a 2 × 2 × 5 repeated-measures ANOVA (α = 0.05). This means that we could not detect small effects of the selected variables. Second, the sample included only young psychology students and was unbalanced in terms of gender (i.e., most of our participants were female between 18 and 30 years of age). Thus, it was not representative of the general population. Since age and gender differences are known to affect the recall of autobiographical memories (St. Jacques and Levine, 2007; Ros and Latorre, 2010; Gardner et al., 2015), additional studies using larger cohorts (see Rouhani et al., 2023) are needed to establish whether our results reflect a general pattern that can be extended to the entire population. Third, the fact that the selected students attended a psychology course held by the project leader might imply a selection bias, since they could easily infer the objective of the study and had at least a general knowledge of the mechanisms underlying memory recall; furthermore, we cannot exclude the possibility that they felt themselves particularly committed to report a large number of events. Lastly, apart from state and trait anxiety, we did not assess the potential impact of COVID-19-related variables and did not include participants who had direct experience with the virus (although it should be noted that there were still few cases at the time in which the study was carried out). Hence, we cannot determine whether the latter factor increased (or decreased) the recall of pandemic-related memories.

Despite these limitations, our results provide preliminary evidence indicating that participants were reasonably accurate in recalling autobiographical events from a relatively short period of time including highly salient events (such as the suspension of all classes after the spreading of the COVID-19 virus and the beginning of the national lockdown) and that the use of external aids was only effective for the furthest day recalled and for events that were not highly distinctive. Some of our findings were well consistent with the general properties of episodic memories (for example, the significant recency effects), while others could be best explained by the distinctiveness and the saliency of the events occurring on specific days. From a practical perspective, these results suggest that asking people to fill Google forms in which they recall the events happened in previous weeks may represent a useful method of contact tracing. Studies on this type of CT paradigm might be useful in medical settings, particularly those involving contagious pathogens.

## Data availability statement

The raw data supporting the conclusions of this article will be made available by the authors, without undue reservation.

## Ethics statement

The studies involving humans were approved by Institutional Review Board of the Department of Psychology -Sapienza University of Roma. The studies were conducted in accordance with the local legislation and institutional requirements. The participants provided their written informed consent to participate in this study.

## Author contributions

CR-A developed the idea for this study. CR-A, SM, and VC drafted the manuscript. PS, AS, FA, AN, MP, and VC contributed conception and designed the study. SM and PS collected the data and organized the database. PS, CR-A, AS, FA, and MP analyzed and interpreted the data. PS, CR-A, VC, and SM contributed to the discussion of content-related issues and to the critical revision of the article and wrote sections of the manuscript. CR-A, SM, and VC wrote the final version of the manuscript. All authors contributed to the article and approved the submitted version.

## Appendix

TABLE A1 Illustrates the means and standard deviations (in parenthesis) of the total number of events reported by participants for the entire period considered (2 weeks, from 27 February to 12 March), divided by category (internal and external events).

**Table:**

Day	AM1			AM2		
	Total	Internal	External	Total	Internal	External
12 March (Day1)	17.1 (11.0)	16.7 (10.7)	0.4 (0.6)	17.0 (10.26)	16.6 (10.0)	0.3 (0.7)
11 March	15.9 (8.9)	15.6 (8.9)	0.3 (0.9)	14.9 (7.94)	14.7 (7.8)	0.1 (0.5)
10 March	15.5 (10.2)	15.1 (9.8)	0.5 (0.9)	13.4 (9.44)	13.1 (9.2)	0.2 (0.5)
9 March (Day2)	13.5 (8.8)	13.4 (8.5)	0.3 (0.7)	12.7 (8.86)	12.4 (8.6)	0.2 (0.5)
8 March	14.9 (8.6)	14.5 (8.5)	0.3 (0.7)	14.8 (8.1)	14.5 (7.9)	0.2 (0.7)
7 March	17.4 (11.0)	16.8 (10.8)	0.5 (0.9)	17.5 (11.9)	17.1 (11.6)	0.3 (0.7)
6 March	16.5 (13.3)	16.1 (12.8)	0.4 (1.1)	17.2 (12.2)	16.7 (11.8)	0.2 (0.7)
5 March (Day3)	14.0 (8.9)	13.6 (8.7)	0.3 (0.7)	14.9 (8.4)	14.7 (8.3)	0.2 (0.6)
4 March (Day4)	16.7 (10.1)	16.0 (9.8)	0.7 (0.8)	16.9 (8.5)	16.3 (8.2)	0.5 (0.8)
3 March	19.0 (10.5)	18.5 (10.2)	0.5 (1.0)	18.5 (8.8)	18.2 (8.4)	0.3 (0.7)
2 March	14.2 (7.5)	14.0 (7.3)	0.1 (0.4)	16.6 (8.3)	16.4 (8.3)	0.1 (0.4)
1 March	9.3 (9.5)	9.3 (9.4)	0.1 (0.3)	13.2 (9.8)	13.0 (9.7)	0.1 (0.4)
29 February	10.8 (8.4)	10.4 (7.9)	0.4 (1.6)	13.8 (7.6)	13.6 (7.5)	0.1 (0.3)
28 February	17.1 (12.5)	16.6 (12.1)	0.5 (0.9)	19.7 (11.4)	19.3 (11.1)	0.4 (0.7)
27 February (Day5)	11.9 (8.3)	11.7 (8.1)	0.2 (0.6)	15.1 (8.0)	14.8 (7.9)	0.2 (0.6)
